# Characteristics of gait pelvic jerk in individuals with femoroacetabular impingement syndrome

**DOI:** 10.1002/jeo2.70373

**Published:** 2025-07-24

**Authors:** Satoshi Machida, Masahiro Tsutsumi, Hajime Utsunomiya, Takuya Ibara, Shintarou Kudo

**Affiliations:** ^1^ Graduate School of Health Sciences Morinomiya University of Medical Sciences Osaka Japan; ^2^ Meirikai Tokyo Yamato Hospital Tokyo Japan; ^3^ Inclusive Medical Sciences Research Institute Morinomiya University of Medical Sciences Osaka Japan; ^4^ Tokyo Research Institute for Sports Medicine Chiba Japan; ^5^ Department of Functional Joint Anatomy, Biomedical Engineering Laboratory Institute of New Industry Incubation, Institute of Science Tokyo Tokyo Japan

**Keywords:** femoroacetabular impingement, gait, patient‐reported outcome measures, pelvic jerk

## Abstract

**Purpose:**

Despite a consensus on gait kinematic changes caused by femoroacetabular impingement (FAI), the characteristics of kinetic changes in patients with FAI remain unclear. Therefore, this study aimed to investigate whether pelvic jerk, which can be assessed by inertial sensors, can detect kinetic differences between individuals with FAI and asymptomatic controls and the association between pelvic jerk and patient‐reported outcome measures in individuals with FAI.

**Methods:**

Thirty patients with FAI and 30 asymptomatic controls participated in this study. To obtain the pelvic jerk time series, all participants walked 10 m at a self‐selected speed using a third lumbar internal sensor. The peak values of the pelvic jerk in the first and second halves of the stance phases (1st‐ and 2nd‐peak pelvic jerks) were also analysed. The patient‐reported outcome measures of individuals with FAI were the international hip outcome tool‐33, hip outcome score‐activities of daily living, and modified Harris hip score.

**Results:**

The FAI group showed lower pelvic jerk than the control group in the first (3%–18% gait cycle) and second halves (42%–54%, 57%–67%) of the stance phase based on the statistical parametric mapping analysis (*p* < 0.05), and also lower peak values corresponding to the respective gait cycles (1st‐peak pelvic jerk, *p* < 0.001; 2nd‐peak pelvic jerk, *p* = 0.002). Multivariate linear regression analysis showed that 1st‐peak pelvic jerk was positively associated with all patient‐reported outcome measures.

**Conclusion:**

Gait kinetic changes in the FAI group were characterised by reduced 1st‐ and 2nd‐peak pelvic jerks. The reduced 1st‐peak pelvic jerk is associated with hip function disability in individuals with FAI. Pelvic jerk may be a simple and quantitative indicator of FAI, although the relevance of this metric must be confirmed in the future studies.

**Level of Evidence:**

Level III, case–control study.

## Graphical abstract

A one‐sentence summary of the key findings. Gait kinetic changes in individuals with femoroacetabular impingement syndrome were characterised by reduced pelvic jerk, which was associated with their hip functional disability.

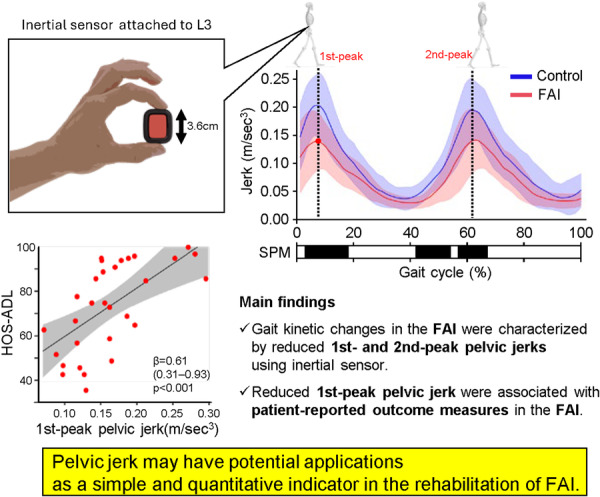

**Abbreviations**
ANCOVA: analysis of covariance
Ax: front‐back component
Ay: left‐right component
Az: vertical component
BMI: body mass index
FABER: flexion abduction external rotation
FADIR: flexion adduction internal rotation
FAI: femoroacetabular impingement
HOOS: hip disability and osteoarthritis outcome score
HOS‐ADL: hip outcome scale‐activities of daily living
iHOT: international hip outcome tool‐33
L3: third lumbar vertebra
LCE: lateral centre‐edge
mHHS: modified Harris hip score
PROMs: patient‐reported outcome measures
SPM: statistical parametric mapping

## INTRODUCTION

Femoroacetabular impingement (FAI) syndrome, characterised by pathological contact between the proximal femur and the acetabulum during hip motion, is recognised as a cause of hip pain, functional limitations during activities of daily living and sports, and reduced quality of life [12, 20, 22]. Structural deformities of FAI can damage intraarticular structures; thus, FAI is also recognised as a common precursor of hip osteoarthritis [5, 11, 17]. FAI is considered a motion‐related clinical disorder of the hip, with a triad of symptoms, clinical signs, and imaging findings [12, 32].

The importance of gait analysis in daily functional activities and return to sports activities is generally recognised [9, 33]. Numerous studies have analysed the gait characteristics of individuals with FAI [3, 6, 9, 13, 16, 21, 23, 25, 27, 30, 33, 34, 35, 36, 38, 42]. Regarding kinematics, two systematic reviews and meta‐analyses have consistently concluded that participants with FAI have a reduced sagittal plane hip range of motion and peak hip extension angles during gait compared with the control group [24, 44]. Regarding kinetics aspects, some previous studies have reported significantly reduced hip moments in individuals with FAI [16, 23, 30, 35, 38, 42], while others found no differences between individuals with FAI and controls [3, 6, 9, 13, 21, 25, 33, 34]. Consequently, the most recent systematic review did not reach a conclusion on kinetic differences in FAI [44], and it remains unclear whether individuals with FAI and controls differ in their gait kinetics. Even in studies reporting differences in kinetics, the movement planes in which individuals with FAI and controls exhibited differences (sagittal [16, 23, 30, 35, 38], frontal [38, 42] and transverse planes [16, 42]) varied. Moreover, few studies have examined whether kinetic features during gait are associated with hip function related to patient‐reported outcome measures (PROMs) [23, 35]. Therefore, if gait kinetics evaluations integrating multiple planes could be easily performed, data accumulation in clinical situations would make it possible to identify whether individuals with FAI have differences in gait kinetics from controls and whether its features are associated with PROMs.

In contrast to the gold standard for kinetic analysis using three‐dimensional motion analysis, inertial sensors can be easily used in clinical situations in terms of space and cost. Jerk, which can be assessed using inertial sensors, is the time derivative of acceleration and reflects joint movement smoothness and changes in force [14, 15, 18]. A recent study clarified that pelvic jerk integration in the sagittal, frontal, and transverse planes can detect gait characteristics after hip trochanteric fractures [18]. Therefore, pelvic jerk analysis may reveal the kinetic characteristics of individuals with FAI during gait.

This study aimed to investigate whether pelvic jerk can detect kinetic differences between individuals with FAI and asymptomatic controls, as well as to evaluate the association between pelvic jerk and PROMs in individuals with FAI. We hypothesised that gait pelvic jerk would differ significantly between individuals with FAI than in asymptomatic controls and would be significantly associated with PROMs.

## MATERIAL AND METHODS

### Study design and participants

Thirty participants diagnosed with FAI (and scheduled to undergo hip arthroscopy) and 30 asymptomatic controls participated in this cross‐sectional study. The study was conducted in accordance with the principles of the Declaration of Helsinki, and the protocol was approved by the ethics committee of Morinomiya University of Medical Sciences (approval number: 2022‐089). The purpose and objectives of the study were explained to the participants in writing, and their informed consent was obtained.

**Figure 1:**
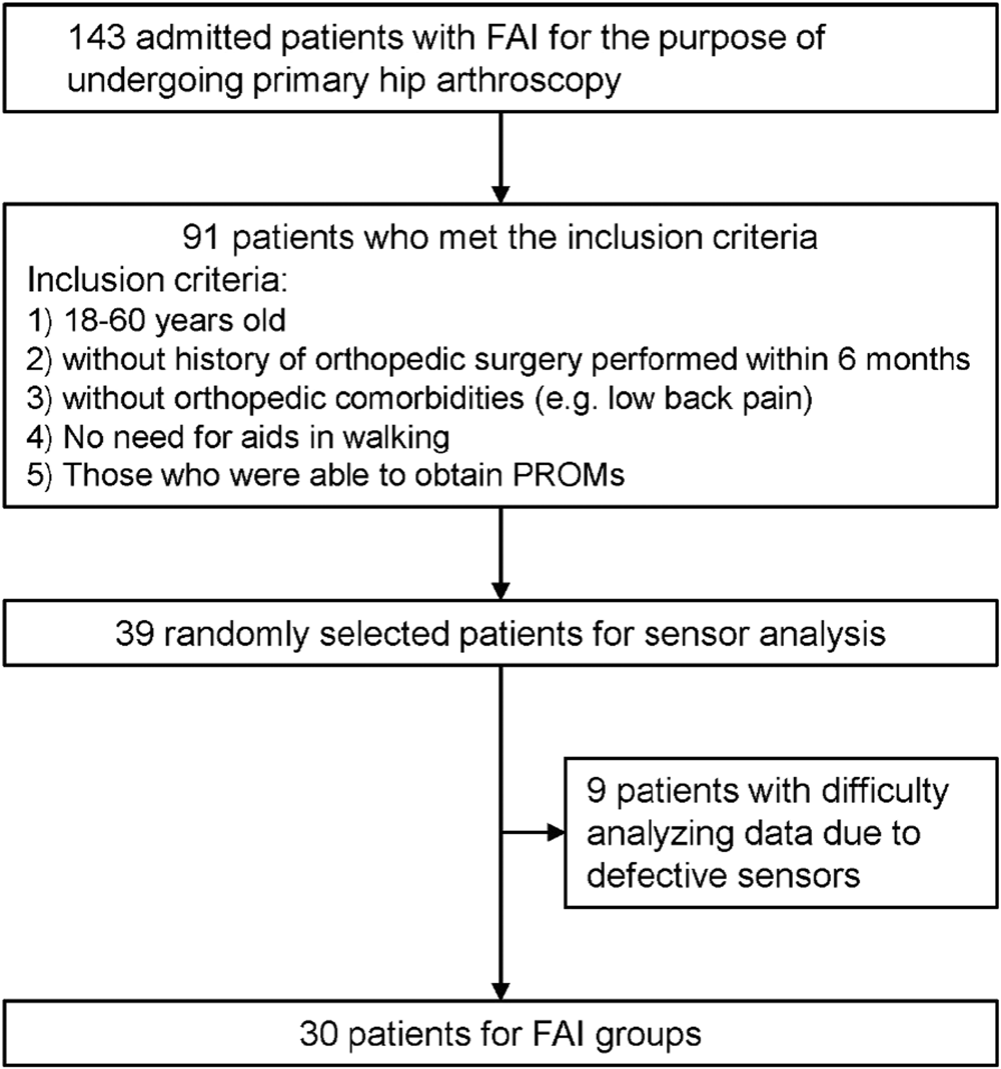
Flow chart for the FAI group. FAI, femoroacetabular impingement; PROMs, patient‐reported outcome measures.

All data were collected from the local hospital between November 2022 and June 2023. Data were extracted from 143 patients with FAI who were admitted for primary hip arthroscopy. The diagnosis of FAI was based on a combination of imaging and clinical physical findings, including flexion adduction internal rotation (FADIR) and flexion abduction external rotation (FABER) tests, by a senior orthopaedic surgeon [43]. The imaging eligible criteria for primary hip arthroscopy owing to FAI included one of the following: (1) an alpha angle of >55° and a lateral centre‐edge (LCE) angle of >25°, (2) LCE angle of >30° and acetabular index of <0°, or (3) LCE angle of >40° [43]. Of the 143 patients admitted with FAI, 91 met the following inclusion criteria: (1) aged 18–60 years, (2) no history of orthopaedic surgery within the past 6 months, (3) no orthopaedic comorbidities (e.g., low back pain), (4) no need for walking aids and (5) ability to complete PROMs. Regarding PROMs, the International Hip Outcome Tool‐33 (iHOT), Hip Outcome Score‐Activities of Daily Living (HOS‐ADL), and modified Harris hip score (mHHS) were recorded [12, 28]. Considering the possibility of measurement failure due to defective inertial sensors, 39 patients (18 males and 21 females; mean age, 37.2 ± 12.3 years) were randomly selected from the 91 eligible patients for analysis. Following the exclusion of nine patients owing to sensor defects, 30 participants (14 males and 16 females; age range, 18–58 years) were assigned to the FAI group (Figure 1). Thirty asymptomatic controls (14 males and 16 females; age range, 22–58 years), reflecting the age distribution and sex ratio of FAI groups as closely as possible, were recruited from the hospital community. These asymptomatic controls met the following inclusion criteria: (1) aged 18–60 years, (2) no history of orthopaedic surgery, (3) no lower extremity or lower back pain and (4) no need for walking aids. Following no exclusion of participants with difficulty analysing data due to sensor defects, all 30 asymptomatic participants were assigned to the control groups.

### Experimental procedure

To obtain data for pelvic jerk during gait, all participants in the FAI and control groups walked 10 m with inertial sensors (Xsens DOT, Movella Technologies, Henderson, NV, USA; size (mm): 30.35 wide × 10.85 deep × 36.30 high; weight: 10.8 g, sampling frequency: 60 Hz) at a self‐selected speed. Pelvic jerk was calculated from pelvic accelerations using the methods described below, and the validity and reliability of the acceleration values obtained by the inertial sensors used in this study have been verified in a previous study [8]. The walking time and the number of steps were also recorded. Inertial sensors were attached to the third lumbar vertebra (L3) [4, 18] and both sides of the femur (the midpoint connecting the greater trochanter and lateral epicondyle). The affected side was used as the start of the gait cycle in the FAI group, whereas the right stance leg was used as the start of the gait cycle in the control group. Four consecutive gait cycles following the fourth step were included in the analysis.

The analysis procedure for pelvic jerk was as follows. First, based on the gait acceleration of the front‐back component (Ax), left‐right component (Ay), and vertical component (Az) recorded with the direction of motion being forward, initial contact was identified on the basis of Az of the pelvis [1], and lower limbs and data were normalised with one gait cycle as 100%. The pelvic accelerations in three directions (Ax, Ay and Az) were time‐differentiated using VENUS3D (Nobby Tech. Ltd., Tokyo, Japan), and their respective jerks were calculated. The jerk was calculated from the first‐time derivative of linear acceleration. The pelvic jerk was defined as the square root of the sum of the squares of the jerk components in each of the three axes [18]. For statistical analysis, the time‐series data of pelvic jerks for the four gait cycles were added and averaged for each participant. A five‐period moving average was calculated to smooth the time‐series data. Additionally, the pelvic jerk time‐series data were divided into the first and second halves of the gait cycle, and their peak values, 1st‐peak pelvic jerk for the first half and 2nd‐peak pelvic jerk for the second half, were identified.

To validate the relationships between pelvic jerk and hip kinetics, nine additional asymptomatic participants (7 males and 2 females; mean age, 26.6 ± 7.3 years; height, 1.7 ± 0.1 m; weight, 62.1 ± 7.4 kg) were recruited and simultaneously assessed for pelvic jerk and hip moment during gait. All participants performed three straight‐line walking trials on a 10‐m level walkway under two cadence conditions (normal, 108 steps/min; [29] fast, 118 steps/min [40]). Pelvic jerk was acquired using the methods described above, and hip moment was measured using a three‐dimensional gait analysis system. The system consisted of nine infrared cameras (sampling frequency, 100 Hz; Vicon, Oxford, UK) and two force plates (sampling frequency, 1000 Hz; AMTI, Watertown, MA, USA). Thirty‐five reflective markers were placed according to the plug‐in gait model. Raw marker trajectory and ground reaction force data were processed using a Butterworth low‐pass filter at 10 and 50 Hz, respectively. Peak values of the external hip flexion moment (during loading response) and extension moment (during terminal stance) were calculated using Nexus (Vicon). The average value from the three trials was used for statistical analysis.

### Statistical analysis

The sample size was determined on the basis of a prior power analysis. Power analysis was conducted using G*Power software and based on a large effect size of *d* = 0.8 [7, 27], *α* = 0.05, and power of 0.80, resulting in a required sample size of 26 participants per group. Considering the possibility of participants dropping out, 60 participants were recruited (30 in each group).

For the basic characteristics (age, sex, height and weight), a two‐sample t‐test or chi‐square test was used to compare data between the groups. Gait speed, step count, and 1st‐ and 2nd‐peak pelvic jerks were compared between the two groups using analysis of covariance (ANCOVA), adjusted for age, sex, and body mass index (BMI), based on a previous study [39]. Additionally, to compare the pelvic jerk waveforms during a gait cycle, a two‐sample t‐test of statistical parametric mapping (SPM) analysis was performed using Python (version 3.11.4; Python Software Foundation, Wilmington, DE, USA). Furthermore, multivariable linear regression analyses were performed to examine the associations between each PROM and the gait variables (gait speed and 1st‐ and 2nd‐peak pelvic jerks) in the FAI group after adjusting for age, sex, and BMI. All significance levels of the comparisons were set at 0.05, and all statistical analyses other than the comparison of the pelvic jerk waveforms were performed using SPSS software (version 27.0; IBM Corp., Armonk, NY, USA).

**Figure 2:**
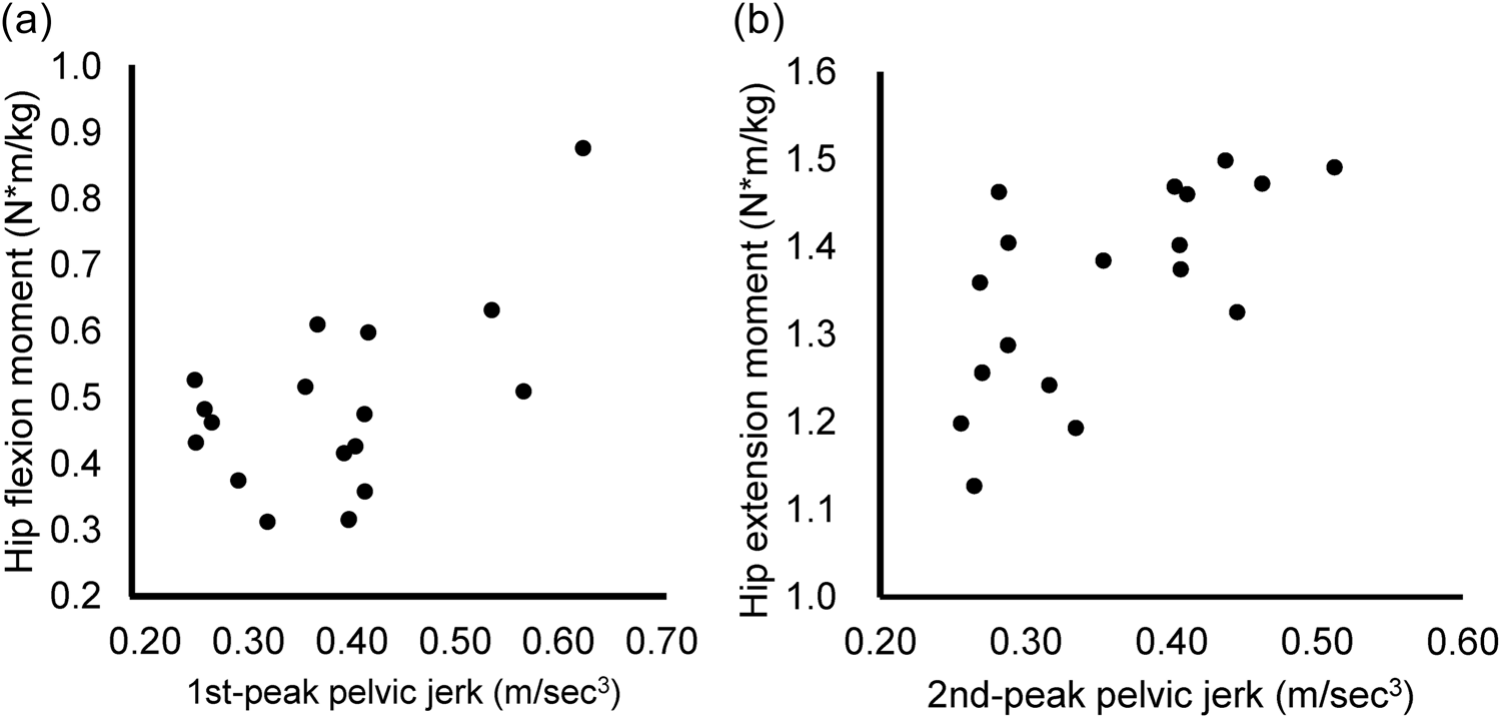
Correlation analysis between pelvic jerk and hip moments. Scatter plots show the relationships between the 1st‐peak pelvic jerk and peak hip flexion moment (a) and between the 2nd‐peak pelvic jerk and peak hip extension moment (b).

To examine the relationships between pelvic jerk and hip moments in the nine additional asymptomatic participants, Pearson correlation analysis was conducted between the 1st‐peak pelvic jerk and peak hip flexion moment and between the 2nd‐peak pelvic jerk and peak hip extension moment. The 1st‐peak pelvic jerk showed a significant positive correlation with peak hip flexion moment (*r* = 0.53 [95% confidence interval, 0.09–0.80], *p* = 0.022), and the 2nd‐peak pelvic jerk also showed a significant positive correlation with peak hip extension moment (*r* = 0.65 [0.27–0.86], *p* = 0.003) (Figure 2).

## RESULTS

The FAI and asymptomatic groups did not show significant differences in age and sex distribution, but the FAI group had a higher BMI and weight than the asymptomatic control group (Table 1).

**Table 1:** Characteristics of participants in the FAI and control groups.

	FAI (*n* = 30)	Control (*n* = 30)	*p*‐value
Age (years)	37.6 ± 12.1 (33.1–42.1)	34.6 ± 10.8 (30.6–38.7)	0.315
Sex	M 14, F 16	M 14, F 16	1.000
Height (m)	1.67 ± 0.08 (1.64–1.70)	1.64 ± 0.09 (1.61–1.67)	0.212
Weight (kg)	61.8 ± 10.1 (58.0–65.6)	56.2 ± 10.2 (52.4–60.0)	0.038
BMI (kg/m^2^)	22.1 ± 2.5 (21.2–23.0)	20.8 ± 2.4 (19.9–21.7)	0.036
Alpha angle (°)	64.9 ± 8.5 (61.7–68.1)		
Centre edge angle (°)	37.0 ± 7.6 (34.2–39.9)		
Acetabular roof obliquity (°)	9.1 ± 5.2 (7.1–11.0)		
VCA (°)	42.0 ± 11.5 (37.6–46.5)		
iHOT	53.8 ± 19.6 (46.5–61.2)		
HOS‐ADL	73.3 ± 19.9 (65.9–80.8)		
mHHS	72.8 ± 17.9 (66.1–79.4)		

*Note*: Data are shown as mean ± standard deviation, and 95% confidence intervals are shown in parentheses.

Abbreviations: BMI, body mass index; F, female; FAI, femoroacetabular impingement; HOS‐ADL, hip outcome scale activities of daily living; iHOT, international hip outcome tool‐33; M, male; mHHS, modified Harris hip score; VCA, vertical centre anterior margin angle.

**Figure 3:**
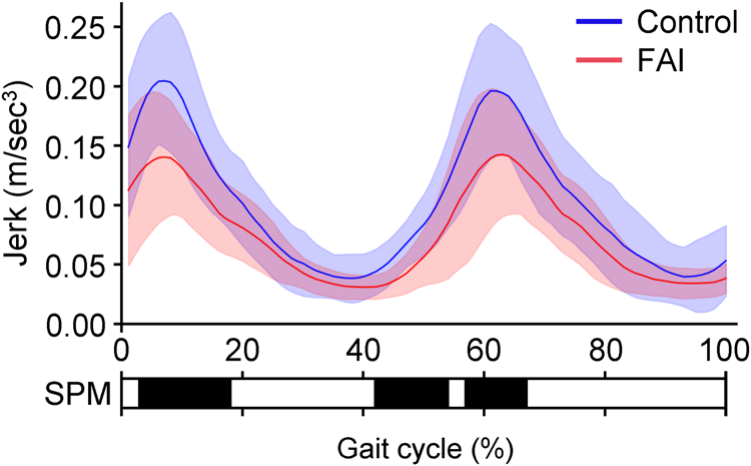
Pelvic jerk waveforms in the FAI and control groups during a gait cycle. A two‐sample t‐test of SPM analysis was performed to compare the mean waveforms with standard deviations between the FAI (red) and control (blue) groups. Intervals with significant differences in the SPM analysis are shown in black. The beginning of the gait cycle (0%) was defined as initial contact, and the percentage of each gait cycle was 100%. FAI, femoroacetabular impingement; SPM, statistical parametric mapping.

Individuals in the FAI group walked more slowly (FAI versus [vs.] controls: 1.11 [95% confidence interval, 1.05–1.16] m/sec vs. 1.32 [1.26–1.37] m/s, *p* < 0.001) and took more steps during the 10‐m walk than those in the control group (FAI vs. controls: 16.9 [16.3–17.4] steps vs. 15.1 [14.6–15.7] steps, *p* < 0.001). Time‐series data of the pelvic jerk showed bimodal waveforms in both groups, with peaks in the first and second halves of the stance phase. Regarding peak values, the FAI group showed lower 1st‐peak (FAI vs. controls: 0.16 [0.14–0.19] m/s^3^ vs. 0.22 [0.20–0.25] m/s^3^, *p* < 0.001) and 2nd‐peak pelvic jerks (FAI vs. controls: 0.17 [0.15–0.19] m/s^3^ vs. 0.22 [0.20–0.24] m/s^3^, *p* = 0.002) than the control group (Table 2). Based on the SPM analysis (Figure 3), the FAI group showed lower pelvic jerk than the controls in the first (3%–18% gait cycle) and second halves (42%–54%, 57%–67% gait cycle) of the stance phase (*p* < 0.05). These intervals with significant differences included the timing of the 1st‐ and 2nd‐peak pelvic jerks.

**Table 2:** Gait parameter data for each group.

	FAI	Control	Mean difference	*p*‐value
Gait speed (m/s)	1.11 (1.05–1.16)	1.32 (1.26–1.37)	−0.19 (−0.27, −0.11)	<0.001
Gait steps (steps)	16.9 (16.3–17.4)	15.1 (14.6–15.7)	1.7 (0.9–2.5)	<0.001
Pelvic jerk (m/s^3^)				
1st peak	0.16 (0.14–0.19)	0.22 (0.20–0.25)	−0.06 (−0.09, −0.02)	<0.001
2nd peak	0.17 (0.15–0.19)	0.22 (0.20–0.24)	−0.05 (−0.08, −0.02)	0.002

*Note*: 95% confidence intervals are shown in parentheses. All parameters are adjusted for age, sex, and body mass index.

Abbreviation: FAI, femoroacetabular impingement.

In multiple regression analysis (Table 3), gait speed and 1st‐peak pelvic jerk were positively associated with iHOT (gait speed; *β* = 0.47, *p* = 0.008, 1st peak; *β* = 0.53, *p* = 0.001), HOS‐ADL (gait speed; *β* = 0.50, *p* = 0.007, 1st peak; *β* = 0.61, *p* < 0.001), and mHHS values (gait speed; *β* = 0.49, *p* = 0.004, 1st peak; *β* = 0.49, *p* = 0.003).

**Table 3:** Relationships between PROMs and gait variables in FAI group.

	Gait speed		1st‐peak pelvic jerk		2nd‐peak pelvic jerk	
	*β*	*p*‐value	*β*	*p*‐value	*β*	*p*‐value
i‐HOT	0.47 (0.13–0.80)	0.008	0.53 (0.23–0.83)	0.001	0.15 (−0.23, 0.53)	0.43
HOS‐ADL	0.50 (0.15–0.86)	0.007	0.61 (0.31–0.93)	<0.001	0.21 (−0.19, 0.62)	0.28
mHHS	0.49 (0.17–0.81)	0.004	0.49 (0.18–0.80)	0.003	0.19 (−0.18, 0.56)	0.30

*Note*: Standardised partial regression coefficients (*β*) are shown with 95% confidence intervals in parentheses.

Abbreviations: FAI, femoroacetabular impingement; HOS‐ADL, hip outcome scale‐activities of daily living; iHOT, international hip outcome tool‐33; mHHS, modified harris hip score; PROMs, patient‐reported outcome measures.

## DISCUSSION

The present study revealed that gait kinetic changes in the FAI group were characterised by reduced 1st‐ and 2nd‐peak pelvic jerks. Notably, the reduced 1st‐peak pelvic jerk was associated with hip function disability, as assessed by PROMs in individuals with FAI. Clinically, rehabilitation for individuals with FAI lacks an objective indicator that incorporates kinetic aspects to guide progression through protocol phases [2, 10, 19]. Pelvic jerk, as measured by inertial sensors, can be easily applied in clinical settings, and its 1st‐ and 2nd‐peaks were correlated with the peak values of external hip flexion and extension moments, respectively. Given the established importance of gait analysis in daily functional activities and return to sports [9, 33], pelvic jerk may serve as a simple kinetic indicator for advancing stages in FAI rehabilitation. The relevance of this metric should be confirmed in future studies.

Regarding the characteristics of FAI gait kinetics compared with controls, Hunt et al. [16] reported reduced peak moments in hip flexion during the early stance phase, and Ng et al. [30] reported reduced peak moments in hip extension during the late stance phase. However, these characteristics were based only on the peak value or local maxima rather than the waveform of the time‐series data, potentially missing differences along the time series (regional focus bias) [31]. A study based on SPM analysis [38] showed reduced hip flexion and adduction moments just before the stance phase, less power by hip flexion during early stance, and less absorbed power by adduction during swing preparation; however, no differences in peak values in the respective gait cycle were reported. In contrast to previous studies, the peak values and time‐series data in this study allowed us to characterise FAI gait kinetics in the first and second halves of the stance phase using pelvic jerk analysis. Based on the correlation between the 1st‐peak pelvic jerk and peak hip flexion moment, the reduced 1st‐peak pelvic jerk in the early stance phase may be associated with the reduced power generation by the hip extensors [37]. Similarly, based on the correlation between the 2nd‐peak pelvic jerk and peak hip extension moment, the reduced 2nd‐peak pelvic jerks in the late stance phase may be related to reduced power generation by the hip flexors [37].

Another novel finding of this study was that the 1st‐peak pelvic jerk in the FAI group was positively associated with PROMs. Regarding the relationships between the kinetic characteristics during the early stance phase and PROMs, Samaan et al. [35] showed a negative association between the hip flexion moment impulse, calculated using the integral of the hip flexion moment, and Hip Disability and Osteoarthritis Outcome Score (HOOS) subscore. Their findings may contradict our results; however, this discrepancy needs to be carefully considered because the HOOS subscore improved postoperatively with an increase in the hip flexion moment impulse in their subsequent study [34]. Therefore, the significance of our positive association between the 1st‐peak pelvic jerk and PROMs requires future consideration, including the postoperative course of the pelvic jerk.

## LIMITATION

This study has some limitations. First, owing to the lack of imaging for the control group, we cannot exclude the possibility that our study risked including individuals with undiagnosed 'silent' FAI, which could have biased the comparison and weakened the validity of our conclusions. Although some previous studies evaluated hip bony morphology using radiographs or magnetic resonance imaging [6, 9, 21, 25, 30, 34, 35, 36, 42], others—like the present study—compared individuals with FAI and asymptomatic controls without imaging of the control group [3, 13, 16, 23, 27, 33, 38]. As these differences in control group imaging did not show any consistent trend in hip kinetics results, the effect of lacking imaging data in our control group may be minimal. Future research should include more rigorous comparisons among symptomatic FAI, asymptomatic FAI, and healthy controls to clarify gait kinetics more precisely. Second, the individuals with FAI whose pelvic jerks were analysed in this study were not subjected to three‐dimensional motion analysis, and the validity of the pelvic jerk values obtained using inertial sensors could not be fully verified. Established tools such as force plates, electromyography, or full‐body motion analysis may provide reference datasets that offer deeper insight into gait abnormalities in FAI. We believe that pelvic jerk may serve as a simple and quantitative indicator of FAI, although its relevance must be confirmed through future studies comparing it with established tool‐based datasets. Third, individuals with FAI exhibited characteristics in the second half of the stance phase; however, the timing of the second half of the stance phase on the affected side was that of the early stance phase on the other side. Therefore, both sides were affected in this study, and the influence of the asymptomatic side cannot be excluded. Fourth, although the statistical comparison was adjusted for age, we cannot rule out the possibility that the approximately 3‐year average age difference between groups may have influenced our findings. Finally, the gait‐jerk characteristics presented in this study did not consider the effects of gait speed. Previous studies have shown that the gait jerk is positively correlated with gait speed [18, 41], and the peak values of the pelvic jerk in this study also showed a positive correlation with gait speed (1st‐peak, Pearson *r* = 0.61 [95% confidence interval, 0.42–0.75], *p* < 0.001; 2nd peak, *r* = 0.54 [95% confidence interval, 0.33–0.70], *p* < 0.001). However, many gait kinetic parameters have a nonlinear relationship with gait speed [26], and it is unclear whether it is appropriate to adjust the jerk value by gait speed in a general linear model such as ANCOVA. The continued accumulation of jerk along with gait speed is necessary to characterise jerk in individuals with FAI based on its relationship to gait speed.

## CONCLUSION

Gait kinetic changes in the FAI groups were characterised by reduced 1st‐ and 2nd‐peak pelvic jerks. In particular, reduced 1st‐peak pelvic jerk may be associated with hip function disability in individuals with FAI. Pelvic jerk may be a simple and quantitative indicator of FAI, although the relevance of this metric must be confirmed in future clinical study with more rigorous comparisons.

## AUTHOR CONTRIBUTIONS

**Satoshi Machida**: Conceptualisation; data curation; formal analysis; investigation; methodology; and Writing–original draft. **Masahiro Tsutsumi**: Conceptualisation; formal analysis; project administration; visualisation; and Writing–review and editing. **Hajime Utsunomiya**: Conceptualisation; project administration; supervision; and Writing–review and editing. **Takuya Ibara**: Methodology; validation; and Writing–review and editing. **Shintarou Kudo**: Conceptualisation; project administration; supervision; and Writing–review and editing. All the authors have read and approved the final version of the manuscript.

## CONFLICT OF INTEREST STATEMENT

The authors declare no conflict of interest.

## ETHICS STATEMENT

The study was conducted in accordance with the principles of the Declaration of Helsinki, and the protocol was approved by the ethics committee of Morinomiya University of Medical Sciences (approval number: 2022‐089). The purpose and objectives of the study were explained to the participants in writing, and their informed consent was obtained.

## Data Availability

The data sets used and/or analysed in the current study are available from the corresponding author upon reasonable request.
